# First-Principles Investigation of Helium Incorporation Effects on the Structural Stability and Electrochemical Performance of Thorium-Based Mixed Oxide Nuclear Fuels

**DOI:** 10.3390/ma19183828

**Published:** 2026-09-08

**Authors:** Lin Zhu, Shi Zhao, Ziyu Cheng, Shiqi Sheng, Yibao Liu, Qianglin Wei, Bao-Tian Wang

**Affiliations:** 1 School of Nuclear Science and Engineering, East China University of Technology, Nanchang 330013, China; 2025010070@ecut.edu.cn (L.Z.); 2024100062@ecut.edu.cn (S.Z.); 2024120797@ecut.edu.cn (Z.C.); 2025120771@ecut.edu.cn (S.S.); ybliu@ecut.edu.cn (Y.L.); 2 Engineering Research Center of Nuclear Technology Application, Ministry of Education (East China Institute of Technology), Nanchang 330013, China; 3 Institute of High Energy Physics, Chinese Academy of Sciences (CAS), Beijing 100049, China; 4 Spallation Neutron Source Science Center, Institute of High Energy Physics, Chinese Academy of Sciences (CAS), Dongguan 523803, China

**Keywords:** thorium-based nuclear fuels, helium incorporation, first-principles calculations, electronic structure, elastic properties, thermal expansion

## Abstract

Helium accumulation is a major contributor to swelling, gas release, and mechanical degradation in oxide nuclear fuels under irradiation. This study employs first-principles density functional theory (DFT) to investigate helium behavior in thorium-based mixed oxide (MOX) fuels. A series of (Th_1−*x*_Pu*_x_*)O_2_ and (Th_1−*x*_U_x_)O_2_ solid solutions (*x* = 0, 0.25, 0.5, 0.75, and 1) was constructed, and the corresponding ground-state configurations were determined through total-energy minimization. The effects of 4.167 at.% helium incorporation on structural stability, electronic structure, elastic response, and thermal expansion were evaluated. Helium migration in ThO_2_, PuO_2_, and UO_2_ was further investigated at octahedral interstitial, metal-vacancy, and oxygen-vacancy sites. Positive helium incorporation energies indicated that helium incorporation is energetically unfavorable for all compositions. Vegard-like behavior was preserved for lattice constants and metal–oxygen bond lengths. The 2.06 eV band gap of UO_2_ disappeared after helium incorporation, whereas band-gap variations in most MOX compositions remained below 0.7 eV. Helium reduced the bulk moduli of (Th_0.75_U_0.25_)O_2_ and UO_2_ by 3.75% and 7.94%, respectively. Thermal expansion coefficients followed the order α_L-UO2_ > α_L-PuO2_ > α_L-ThO2_, with α_L_ of UO_2_ nearly doubling. Metal vacancies acted as helium traps, whereas adjacent oxygen vacancies provided the lowest migration barrier of 0.42 eV. These results indicate that increasing ThO_2_ content improves the resistance of MOX fuels to helium-induced degradation.

## 1. Introduction

Uranium-based fuels have historically dominated commercial nuclear power generation, with pressurized water reactors (PWRs) using UO_2_ as fuel accounting for nearly four-fifths of the reactors worldwide [1]. However, after decades of mining and consumption, currently identified uranium resources are expected to sustain present demand for only about one century [2]. In this context, thorium-based fuels have been regarded as promising alternatives because thorium is approximately three times more abundant than uranium [3]. ThO_2_, the most stable oxide of thorium, exhibits excellent chemical inertness and thermal stability [3,4]. Incorporating ThO_2_ into UO_2_-based mixed oxide (MOX) fuels can suppress further oxidation of uranium and improve the chemical stability of the nuclear fuel [3]. Although the cost of the homogeneous thorium–uranium fuel cycles is approximately 20–30% higher than that of the equivalent purely uranium fuel cycles [5], it can significantly reduce the production of long-lived light actinides and plutonium [6]. In particular, the production of plutonium in thorium-based cycles is only approximately one-third of that in the traditional uranium cycles [4].

Compared with traditional uranium UO_2_ fuel, thorium-based MOX fuel has several advantages. The melting point of ThO_2_ is approximately 3400 °C [7], which is significantly higher than that of UO_2_ (≈2800 °C) [8], indicating that it has better stability at high temperatures. Thorium by itself is not fissile; however, it can be transformed into ^233^U, which is fissile through neutron capture, thereby facilitating continuous extraction of nuclear energy [9]. As ThO_2_, UO_2_, and PuO_2_ have fluorite structures and share similar physical properties, continuous solid solutions can be formed by substituting Th, U, and Pu in the cation sublattices [10]. This structural compatibility is crucial for the fabrication of thorium-based MOX fuels, ensuring phase stability and reliable in-reactor performance, particularly under high-burnup conditions involving prolonged irradiation and high fission-product accumulation [11].

The retention of inert gases due to irradiation is a key criterion for evaluating nuclear fuel performance. Helium (He) is produced through the α-decay of actinides, ternary fission, and the ^17^O(n,α)^14^C reaction [12]. It is also used as the inert gas in the pellet-cladding gap, that is, between fuel pellets and cladding, rendering it one of the most important gases in fuel rods. Because of its high mobility in oxide fuels [13], He can diffuse into interstitial sites, vacancies, and defect clusters. During extended operation or storage, He may accumulate at grain boundaries or defect clusters, forming pressurized bubbles that degrade grain cohesion, promote swelling, and reduce mechanical integrity. Molten salt stack structure alloys also suffer from issues such as helium embrittlement caused by neutron irradiation [14].

Owing to the radiotoxicity of uranium and plutonium and the associated challenges in handling these materials, experimental studies on the behavior of He in Th-MOX fuel are limited. Therefore, ab initio calculations based on density functional theory (DFT) provide an effective approach for investigating the effects of He in nuclear fuels on the atomic scale. More broadly, recent first-principles studies of chemically asymmetric low-dimensional systems have confirmed that DFT can establish a correlation between structural stability, lattice distortion, and electronic response [15,16].

The linear 1*k*-antiferro magnetic (1*k*-AFM) configuration was adopted for UO_2_ as an approximation to its true paramagnetic order, which has been proven to be a reasonable approximation of its magnetic behavior [17]. Recently, DFT studies have also used the 1*k*-AFM configuration to investigate the structural stability, electronic, and mechanical properties of PuO_2_, UO_2_, and MOX solid solutions [18,19,20]. In one study [21], multiple helium atoms were sequentially doped into a ThO_2_ supercell, where lattice distortion was observed when the He concentration exceeded 4.167 at%. Accordingly, this doping level is considered to represent an extreme condition.

The trapping, aggregation, and ordering behavior of helium atoms in thoria (ThO_2_) was investigated using a combination of ion beam irradiation experiments and first-principles calculations [22]. Shao et al. compared the stability of various inert gases at different positions in ThO_2_ and also investigated the clustering behavior of helium atoms in ThO_2_ [23,24]. Rahman et al. demonstrated that the thermal conductivity of ThO_2_ is strongly dependent on the fission products (FPs) concentration and vacancies in the temperature range 300–1500 K [25,26], highlighting the importance of point defect–fission product interactions in governing its heat transport performance. Although these studies have provided important insights into helium trapping, aggregation and site stability in ThO_2_, the influence of helium incorporation on the electronic, mechanical, and thermodynamic properties of (Th_1−x_Pu_x_)O_2_ and (Th_1−x_U_x_)O_2_ remains insufficiently understood.

In this study, the projector-augmented wave (PAW) [27] framework was employed in DFT calculations to investigate the behavior of helium incorporation in Th-MOX fuels. A series of 2 × 1 × 1 supercell models of (Th_1−x_Pu_x_)O_2_ and (Th_1−x_U_x_)O_2_ with *x* = 0, 0.25, 0.5, 0.75, and 1 were constructed. Different ThO_2_ fractions, local cation distributions, and collinear 1*k*-AFM configurations were considered, and the ground-state structures were determined by total-energy minimization. The effects of helium incorporation (with a doping concentration of 4.167 at.%) on lattice parameters, electronic structure, elastic constants, and thermal expansion were systematically investigated. Helium migration behaviors in ThO_2_, PuO_2_ and UO_2_ are further investigated considering interstitial sites, metal vacancies (V_m_) and oxygen vacancies (V_o_).

## 2. Calculation Method and Models

### 2.1. Calculation Method

The (Th_1−x_M_x_)O_2_ models, where M = Pu/U, were constructed from the 12-atom conventional fluorite Fm-3m cell containing four cations and eight oxygen atoms. A two-times supercell was generated by doubling the conventional cell along the [100] direction. Each pristine supercell therefore contained eight cation sites and sixteen oxygen atoms, giving 24 host atoms. For x = 0, 0.25, 0.50, 0.75 and 1, the corresponding supercells were (Th_8−n_M_n_)O_16_, where M = Pu/U, *n* = 0, 2, 4, 6 and 8, respectively. For the intermediate compositions, symmetry-distinct cation arrangements were relaxed, and the lowest-energy configuration among those tested was selected. The same cation arrangement was used for each pristine and He-containing energy pair.

For the U and Pu containing structures, the magnetic state was initialized using a collinear 1*k*-antiferromagnetic arrangement on the U or Pu sites. The magnetic cations within each (100) plane were assigned parallel moments. The initial magnetic moments were Pu (±3.72 ${µ}_{B}$) [28], U (±1.74 ${µ}_{B}$) [29], where the ± signs denote antiparallel collinear magnetic moments. For Th, O, and He were initialized with 0 magnetic moments.

DFT calculations were performed using the Vienna Ab initio Simulation Package (VASP, version 5.4) [30,31]. The electronic exchange–correlation interaction was described within the generalized gradient approximation (GGA) as formulated by Perdew, Burke, and Ernzerhof (PBE) [32]. The interactions between valence electrons and ionic cores were described within the PAW [27,33] formalism using standard scalar-relativistic datasets. The following electron-states were treated as valence states: Th (6*s*^2^6*p*^6^5*f*^1^6*d*^1^7*s*^2^), Pu (6*s*^2^6*p*^6^5*f*^4^6*d*^2^7*s*^2^), U (6*s*^2^6*p*^6^5*f*^2^6*d*^2^7*s*^2^), and O (2*s*^2^2*p*^4^).

Collinear spin polarization was treated within the spin-density-functional framework [34]. The 1*k* antiferromagnetic configurations were initialized by assigning antiparallel magnetic moments to the Pu/U sublattices, after which the spin densities were optimized self-consistently. Explicitly, the spin–orbit coupling (SOC) and noncollinear magnetism were not included. Although SOC is important for certain properties of heavy-metal compounds, it has been numerically found [19,35,36,37] and physically analyzed [38,39] that the inclusion of the SOC has little effect on the bulk and one-electron properties of ThO_2_, PuO_2_ and UO_2_.

The Hubbard model was integrated in the form of Liechtenstein formalism, owing to the strong Coulomb repulsion originating from the 5*f* electronic interactions among Th, U and Pu [40]. The values of Coulomb energy (*U*) and the exchange energy (*J*) were respectively set to 4.5 and 0.5 eV for thorium [41,42,43], 4.7 and 0.7 eV for plutonium [17,18,19] and 4.5 and 0.51 eV for uranium [17,44] cations. These values are consistent with the parameterization values adopted in previous MOX calculations [45]. The electron wave functions were expanded in a plane-wave basis set with a cutoff of 550 eV. Full structural relaxations were performed until the Hellmann–Feynman forces on all atoms were less than 0.02 eV Å^−1^.

**Figure A1:**
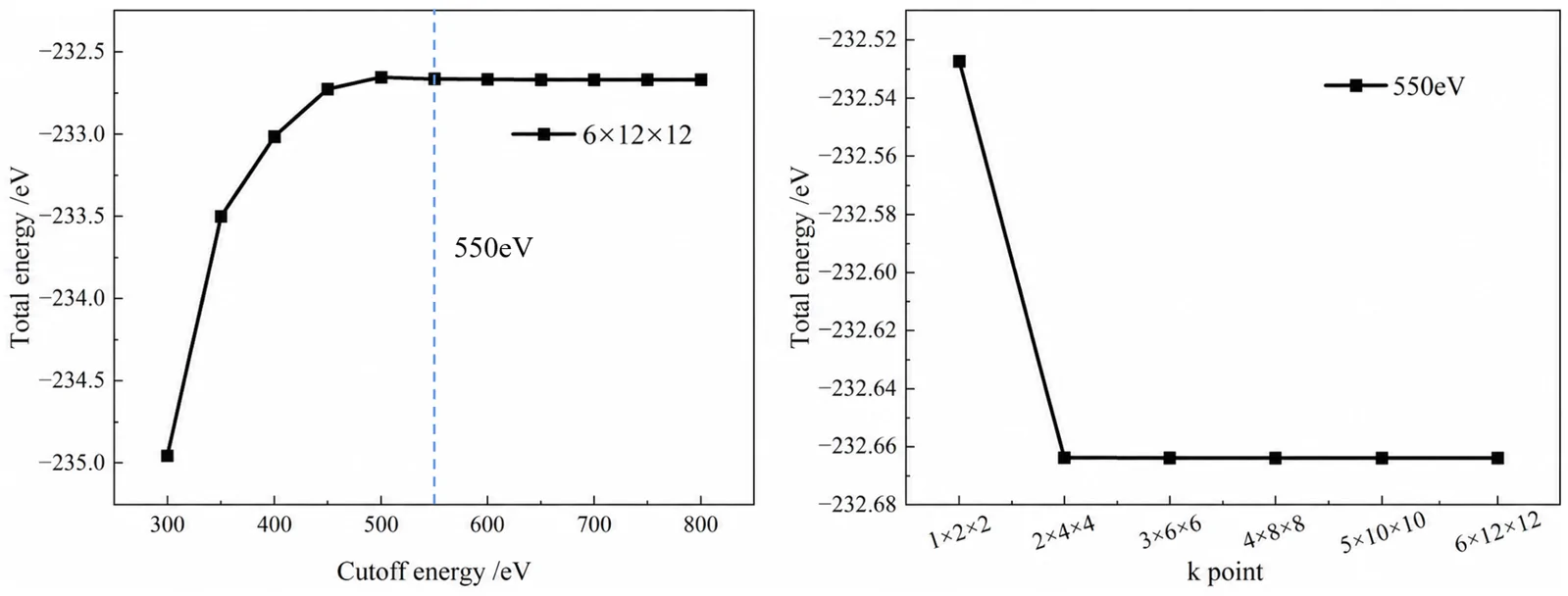
Convergence of total energy as a function of plane-wave cutoff energy and k points. The vertical blue dashed line marks the plane-wave cutoff energy of 550 eV adopted for subsequent calculations.

Brillouin-zone sampling was performed using a 4 × 8 × 8 *k*-point mesh generated using the Gaussian smearing method. The convergence tests for the plane-wave cutoff energy and k-point mesh are presented in Appendix A (Figure A1). For each composition (*x* = 0, 0.25, 0.5, 0.75, 1), the ground-state structures of the mixed-oxide solid solutions were obtained through total-energy minimization. Helium atoms were subsequently introduced into selected sites, and the final optimized configurations were determined.

The elastic constants were calculated for the fully relaxed pristine and He-containing 2 × 1 × 1 supercells using the stress–strain method. Starting from the equilibrium structures, six linearly independent strain patterns were applied with strain amplitudes of ±0.5%. For each strained configuration, the strained lattice vectors were held fixed, while the internal atomic coordinates were relaxed until the maximum residual force on each atom was below 0.02 eV Å^−1^. All reference structures were relaxed without symmetry constraints.

The temperature-dependent thermal expansion properties were calculated using the phonon-based quasi-harmonic approximation (phonon-QHA). For each composition (*x* = 0, 0.25, 0.5, 0.75, 1), volumes spanning −3% to +3% relative to the equilibrium volume were generated. At each fixed volume, the internal atomic coordinates were fully relaxed while the cell shape was constrained, using an energy cutoff of 550 eV, a 4 × 8 × 8 electronic *k*-point mesh and convergence thresholds of 10^−7^ eV and 0.02 eV Å^−1^. The harmonic interatomic force constants were obtained using the finite-displacement method. A 2 × 1 × 1 phonon supercell and an atomic-displacement amplitude of 0.01 Å were employed.

At each volume and temperature, the total Helmholtz free energy was evaluated as

(1) $$F\left(V,T\right)=E_{DFT}\left(V\right)+F_{vib}\left(V,T\right)$$

where $E_{DFT}\left(V\right)$ is the static DFT total energy at volume *V* and $F_{vib}\left(V,T\right)$ is the harmonic vibrational Helmholtz free energy given by

(2) $$F_{vib}\left(V,T\right)=\sum_{q\nu }\left[\frac{1}{2}\hbar \omega_{q\nu }\left(V\right)+k_{B}T\ln\left(1-\exp\left[-\frac{\hbar \omega_{q\nu }\left(V\right)}{k_{B}T}\right]\right)\right]$$

where $\omega_{q\nu }\left(V\right)$ denotes the frequency of phonon mode (q,ν), $k_{B}$ is the Boltzmann constant, and ℏ is the reduced Planck constant. At each temperature, the calculated $F\left(V,T\right)$ values were fitted as a function of volume, and the zero-pressure equilibrium volume $V_{eq}\left(T\right)$ was determined from

(3) $${\left(\frac{\partial F(V,T)}{\partial V}\right)}_{T}=0$$

The volumetric thermal expansion coefficient was subsequently evaluated as

(4) $$\alpha_{V}\left(T\right)=\frac{1}{V_{eq}\left(T\right)}{\left(\frac{\partial V_{eq}\left(T\right)}{\partial T}\right)}_{p}$$

and the equivalent isotropic linear thermal expansion coefficient was obtained from

(5) $$\alpha_{L}\left(T\right)=\frac{\alpha_{V}\left(T\right)}{3}=\frac{1}{{3V}_{eq}\left(T\right)}{\left(\frac{\partial V_{eq}\left(T\right)}{\partial T}\right)}_{p}$$

The present QHA treatment accounts for the temperature dependence associated with lattice vibrations and the volume dependence of the phonon frequencies. Electronic thermal excitations, magnetic and configurational entropies, and explicit phonon–phonon anharmonic effects beyond the QHA were not included.

The reference energy of an isolated He atom was calculated using a 1 × 1 × 1 *k*-point mesh. The minimum-energy migration pathways of He in ThO_2_, PuO_2_, and UO_2_ were determined using the climbing-image nudged elastic band (CI-NEB) method. For each migration pathway, the fully relaxed initial and final configurations were connected by a series of intermediate images. During the CI-NEB optimization, the supercell volume was fixed at that of the relaxed initial configuration, while the atomic coordinates of each image were optimized subject to the NEB constraints. The He migration energy barrier was calculated as

(6) $$\Delta E_{m}=E_{TS}-E_{IS}$$

where $E_{TS}$ and $E_{IS}$ are the DFT total energies of the transition state and the initial state, respectively. Unlike the phonon-QHA calculations used to evaluate thermal expansion, the CI-NEB calculations were performed within the static 0 K DFT framework. Therefore, the reported $\Delta E_{m}$ values represent static migration energy barriers on the 0 K potential-energy surface rather than temperature-dependent migration enthalpies or migration free-energy barriers. Vibrational zero-point-energy corrections, finite-temperature vibrational free-energy contributions, migration entropy, and explicit anharmonic effects were not included in the present CI-NEB calculations.

### 2.2. Calculation Models

**Figure 1:**
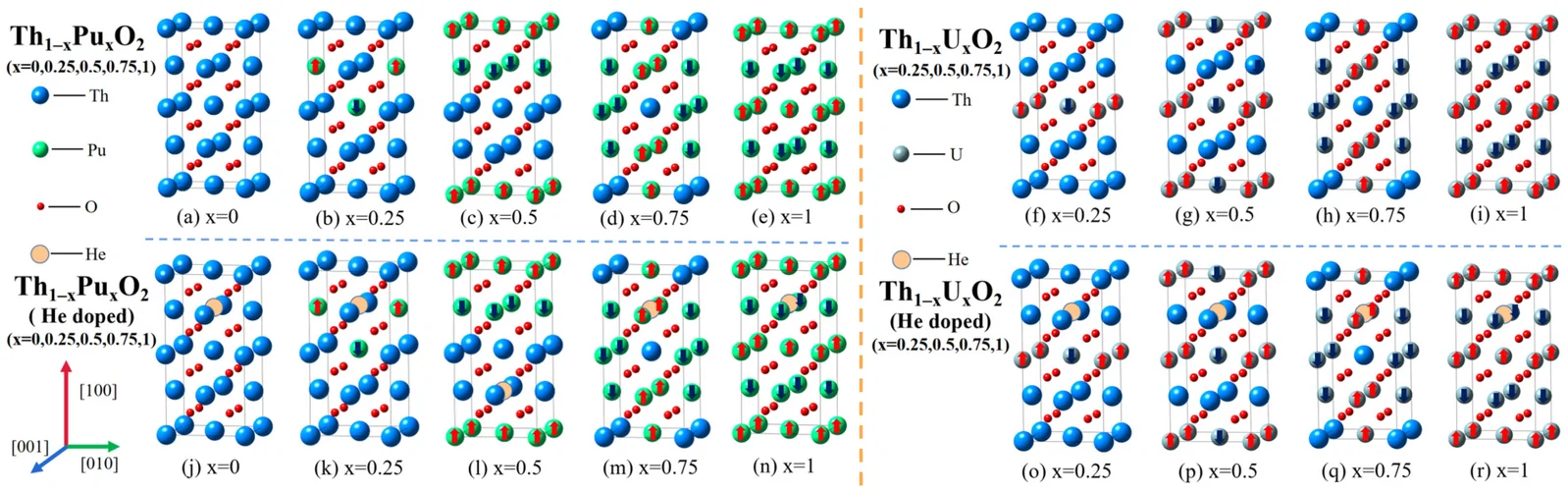
The 2 × 1 × 1 supercell structure and magnetic moment arrangement diagram of (Th_1−x_Pu_x_)O_2_ and (Th_1−x_U_x_)O_2_ (x = 0, 0.25, 0.5, 0.75, 1) before and after He atom doped. (**a**–**e**) (Th_1−x_Pu_x_)O_2_ (x = 0, 0.25, 0.5, 0.75, 1); (**f**–**i**) (Th_1−x_U_x_)O_2_ (x = 0.25, 0.5, 0.75, 1); (**j**–**n**) (Th_1−x_Pu_x_)O_2_ (x = 0, 0.25, 0.5, 0.75, 1) with He doped; (**o**–**r**) (Th_1−x_U_x_)O_2_ (x = 0.25, 0.5, 0.75, 1) with He doped. The red and black arrows indicate antiparallel orientations along [100] direction.

The crystal models employed in this study are illustrated in Figure 1. The red and black arrows denote the opposite spin orientations associated with the 1*k*-AFM configuration. Single helium atoms were initially introduced at three representative sites within the supercell: the face-centered position, the octahedral interstitial sites (OISs), and the midpoint between two nearest neighboring oxygen atoms. Relaxed structures show that an isolated helium atom consistently migrates to the OISs in defect-free mixed oxides: (Th_1−x_Pu_x_)O_2_ and (Th_1−x_U_x_)O_2_. This preference is consistent with earlier calculations and experiments on ideal ThO_2_, PuO_2_ and UO_2_ fluorite lattices [21,46,47]. Therefore, the OIS configuration serves as the reference state for He in vacancy-free lattices.

## 3. Results

### 3.1. Lattice Parameters

**Figure 2:**
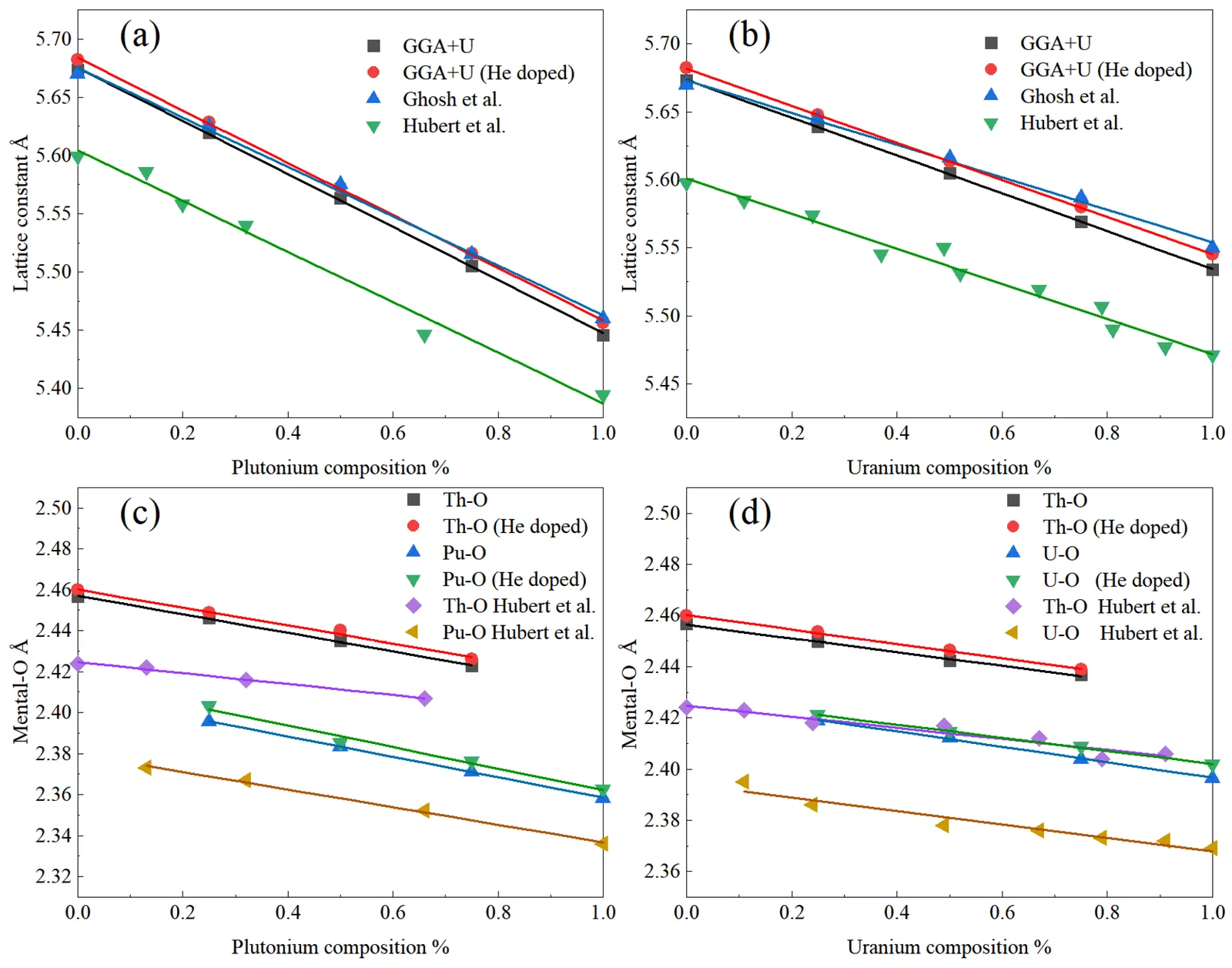
Lattice constant and the average metal–oxygen bond lengths (Å). (**a**) Lattice constant of (Th_1−x_Pu_x_)O_2_ before and after He atom doped [10,45]; (**b**) lattice constant of (Th_1−x_U_x_)O_2_ before and after He atom doped [10,45]; (**c**) the average metal–oxygen bond length of (Th_1−x_Pu_x_)O_2_ before and after He atom doped [10]; (**d**) the average metal–oxygen bond length of (Th_1−x_U_x_)O_2_ before and after He atom doped [10].

The variations in lattice constants and metal–oxygen bond lengths of MOX (Th_1−x_Pu_x_)O_2_ and (Th_1−x_U_x_)O_2_ (*x* = 0, 0.25, 0.5, 0.75, 1) before and after He incorporation are presented in Figure 2. The calculated lattice constant of ThO_2_ is 5.67 Å, which is close to the calculated results reported in other studies [48,49,50,51,52], and higher than the experimental values [53,54,55] by approximately 1.25%, which can be attributed to the limitations of the employed GGA method. For PuO_2_ and UO_2_, considering the AFM order of the f-electrons of Pu and U along the *x* ([100]) direction results in a small difference between the lattice parameters a_0_ and the constants along the *y* ([010]), and *z* ([001]) directions. The AFM configurations of both dioxides lower the crystal symmetry of the fluorite structures from cubic to tetragonal. The calculated a_0_/b_0_ ratio for PuO_2_ and UO_2_ are, respectively, 1.01 and 0.99. Unlike the magnetic transition temperature of 4 K of PuO_2_ predicted by Moli et al. [56], the Néel temperature of UO_2_ (T_N_ = 30.8 K) has been confirmed by experimental measurements [57]. These findings provide theoretical support for the tetragonal phase predicted by DFT calculations at 0 K, which is consistent with previous studies [58,59,60].

Helium incorporation produces only modest volume expansion because of its small atomic size. Across the studied compositions, volume expansion remains below 0.6%. Lattice constants and average metal–oxygen bond lengths retain Vegard-type [61] trends before and after He incorporation. The mixed oxides therefore preserve good lattice compatibility across the Th–Pu and Th–U substitution ranges.

For (Th_1−x_Pu_x_)O_2_ containing less than 75%, helium preferentially occupies the Pu-rich regions, resulting in a maximum increase of 0.32% in the average Pu–O bond length. When the ThO_2_ content is below 50%, helium is instead located in the Th-rich region, leading to a maximum increase of 2.10% in the average Th–O bond length. In (Th_1−x_U_x_)O_2_, the effect of helium on the U–O bond length is highly correlated with the UO_2_ content, with the maximum increase in the U–O bond length reaching 2.22% in pure UO_2_.

### 3.2. Incorporation Energy

The influence of helium incorporation on the host lattice can be quantified in terms of the helium incorporation energy. The calculated helium incorporation energy in the ThO_2_ supercell is in good agreement (Table 1) with the previously reported DFT values of 0.75–0.92 eV [21,23]. Similarly, the calculated incorporation energy for the PuO_2_ supercell is consistent with the value of 1.02 eV reported by Gryaznov et al. [62].

For UO_2_, the helium incorporation energy of 1.52 eV is higher than the previously reported theoretically estimated values of 0.98–1.3 eV [42,63]. This deviation can be attributed to the strong sensitivity of the calculated incorporation energy to the supercell size [64]. To verify this dependence, additional calculations were performed using a 2 × 2 × 2 supercell, which reduced the helium incorporation energy in UO_2_ to 1.16 eV.

### 3.3. Density of States

**Figure 3:**
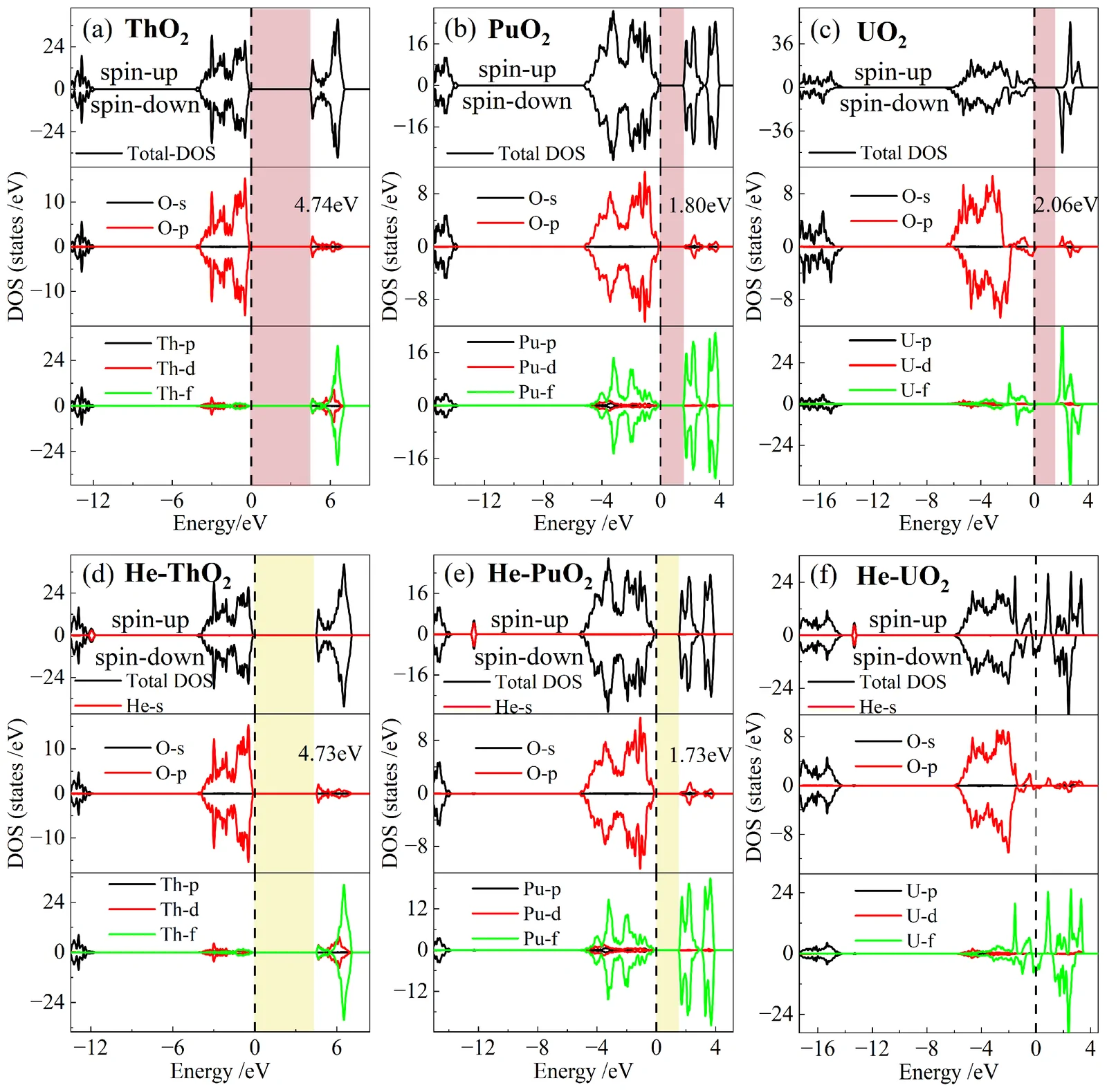
(**a**) Electronic density of states of ThO_2_; (**b**) electronic density of states of PuO_2_; (**c**) electronic density of states of UO_2_; (**d**) electronic density of states of ThO_2_ with He doped; (**e**) electronic density of states of PuO_2_ with He doped; (**f**) electronic density of states of UO_2_ with He doped. The upper part above the horizontal zero line is spin-up, the lower part is spin-down, and the vertical dashed line indicates the Fermi level. The light-red and light-yellow shaded regions, together with the corresponding numerical values, denote the band gaps of the pristine and He-incorporated systems, respectively.

Compared with pure ThO_2_, which has a band gap of approximately 4.74 eV (Table 2), all MOX compositions exhibit band gaps below 3 eV. With increased helium incorporation, the band gaps of each component decreased; however, the reduction was within 0.7 eV. Figure 3 shows the partial and total density of states (DOS) of ThO_2_, PuO_2_ and UO_2_ before and after helium incorporation. In pure ThO_2_, the top of the valence band is primarily composed of O 2p states; the unoccupied states are mainly due to the Th 5f and 6d orbitals (Figure 3a). Significant overlap exists between the O 2s and Th 6p states in the deeper valence band region, consistent with pronounced orbital hybridization. The He 1s states lie approximately 7.7 eV below the O 2p level, with an overlap limited to about 0.3 eV with the O 2s states. This limited overlap indicates a weak direct electronic interaction between the interstitial He and the surrounding oxide matrix.

The DOS of PuO_2_ reveals two prominent peaks: one dominated by Pu 5f states near −3.23 eV and another dominated by O 2p states at approximately −1.09 eV. The saddle region within this range is contributed by O 2p and Pu 5f hybridization, indicating strong mixing between O 2p and Pu 5f states, resulting in covalency, which is different from that of ThO_2_ and UO_2_. The DOS of each MOX (Th-Pu) can be approximated as a superposition of the DOS of ThO_2_ and PuO_2_. The Pu 5f states are distributed within the band gap region of ThO_2_, consistent with that reported in previous studies. This behavior is one of the primary reasons for the observed band-gap reduction in MOX fuels.

The electronic structure of UO_2_ shares many similarities with that of ThO_2_, particularly in that the unoccupied states are predominantly governed by the 5f orbitals. However, UO_2_ exhibits a narrower conduction band, with a width of approximately 2.71 eV. In contrast to that in ThO_2_, the O 2p-dominated states in the valence band of UO_2_ are located farther from the Fermi level and shift toward lower energies as the UO_2_ fraction increases in Th-U MOX systems. In addition, a narrow band appears in the energy range of −1.76 to −0.12 eV, originating from hybridization between U 5f and O 2p states. In (Th_1−x_U_x_)O_2_, the width of this narrow band increases monotonically with increasing UO_2_ content, whereas helium incorporation exerts only a minor influence on its width. Notably, in helium-incorporated UO_2_, the Fermi level shifts to the finite DOS region, indicating that the introduction of helium causes pure UO_2_ to lose its inherent insulating properties.

### 3.4. Elastic Properties

To determine the effect of helium incorporation on the elastic properties of Th-MOX, we calculated the elastic constants for both the original and helium-doped configurations and comprehensively compared the GGA + U calculations with previous ab initio calculations and experimental values (Table 3). For helium-incorporated systems, maintaining complete crystal symmetry may mask local mechanical responses induced by impurities. Therefore, symmetry constraints were removed in our calculations to capture independent directional responses. Consequently, nine independent elastic constants were obtained for all the systems considered except ThO_2_.

Within small homogeneous strains, the elastic constants were obtained using the central finite-difference relation given in Equation (7):

(7) $$C_{ij}=\frac{\sigma_{i}\left(+\delta e_{j}\right)-\sigma_{i}\left(-\delta e_{j}\right)}{2\delta }$$

Here, (*i*, *j* = 1, 2, 3, 4, 5, 6) label the stress and strain components; in Voigt notation, respectively, δ = 0.005 is the dimensionless strain amplitude, and $e_{j}$ is the (*j*)th unit basis vector in the six-dimensional strain space. Accordingly, $\sigma_{i}\left(+\delta e_{j}\right)$ and $\sigma_{i}\left(-\delta e_{j}\right)$ denote the (*i*)th components of the stress tensors calculated for configurations subjected to positive and negative strains, respectively, in the (*j*)th strain component. Consequently, the reported elastic constants represent the relaxed-ion elastic response.

For ThO_2_, although the C_11_ value calculated by GGA + U is about 20 GPa less than the experimental value of 367 GPa reported in previous studies [45,48,75,76], the calculated C_12_ and C_44_ values are in good agreement with the experimental measurements of Mecedo et al. [77]. Because PuO_2_ and UO_2_ exhibit a tetragonal crystal structure due to 1*k*-AFM, our calculated value of C_11_ is 3.72% higher and 2.77% lower, respectively, than that of C_22_. The C_12_ value of PuO_2_ overestimates the previously reported value [20,45,78] by only 4.64%, indicating good agreement with the literature. For UO_2_, although the GGA + U calculated elastic constants agree well with previous theoretical values, C_11_ and C_12_ are slightly underestimated (by 2.75–8.93%), consistent with earlier GGA + U studies [79,80], suggesting a systematic method-related offset.

**Figure 4:**
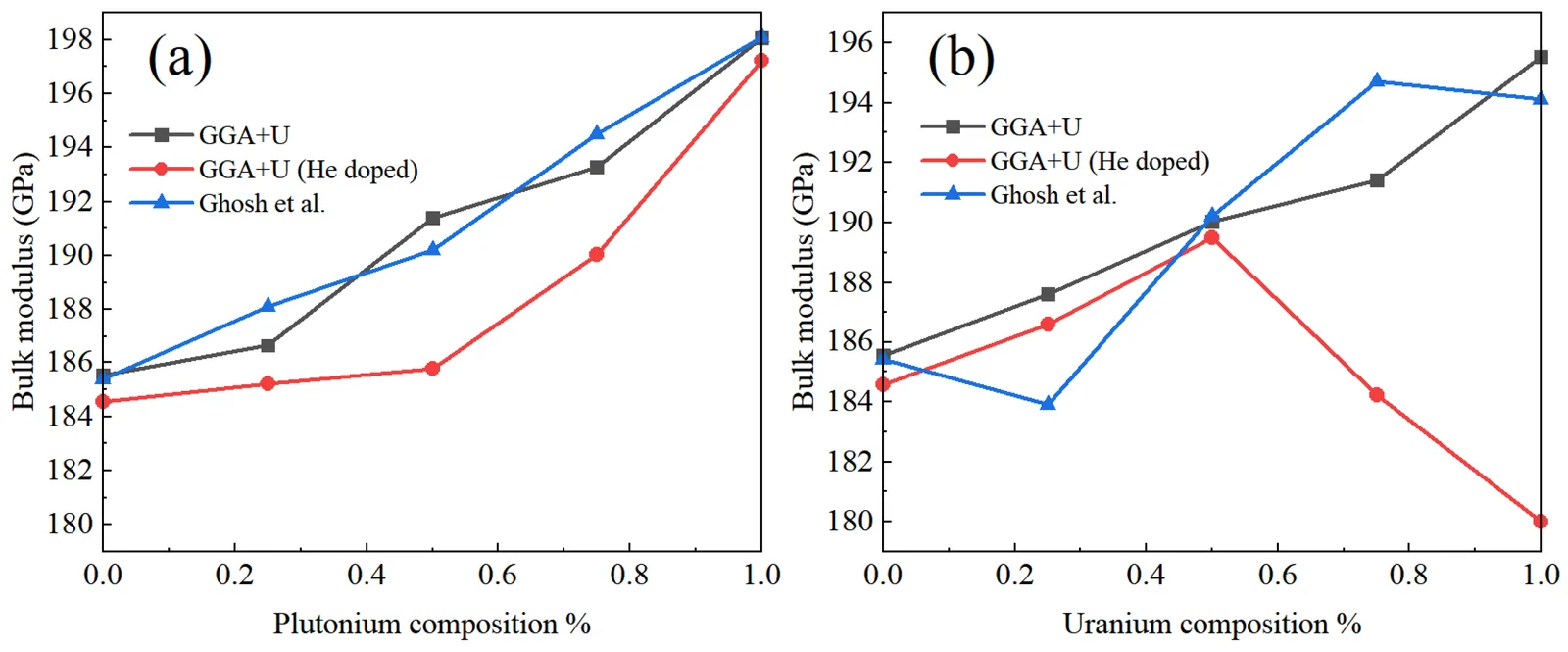
The bulk modulus of two mixed oxides before and after He doped [45]. (**a**) The bulk modulus of (Th_1−x_Pu_x_)O_2_ mixed oxides before and after He doped; (**b**) the bulk modulus of (Th_1−x_U_x_)O_2_ mixed oxides before and after He doped.

The bulk modulus (B) of (Th_1−x_Pu_x_)O_2_ and (Th_1−x_U_x_)O_2_ before and after helium incorporation is shown in Figure 4. The calculated trends are consistent with the bulk moduli of (Th_1−x_Pu_x_)O_2_ mixed oxides obtained by Ghosh et al. [45] using a primitive unit cell. The mechanical properties of both MOX systems lie between those of their two oxides and vary nearly linearly with composition, indicating good lattice compatibility and solid-solution behavior. As the C_11_ and C_12_ values calculated by GGA + U underestimate experimental data, the bulk modulus B (193–198 GPa) also underestimates the experimental value of ThO_2_ by approximately 5%.

Compared with Th-Pu MOX, (Th_0.25_U_0.75_)O_2_ and UO_2_ exhibit greater helium-induced reductions in bulk modulus, decreasing by approximately 7.18 GPa and 15.54 GPa, respectively. This phenomenon can be explained from two perspectives. Helium is barely dissolved into the crystal lattice in UO_2_ (low solubility), as reported in Refs. [82,83]. The maximum helium concentrations measured in UO_2_ specimens have been demonstrated to be less than 1.1 at.% in experimental characterizations [84,85,86]. Simulations conducted using a model with a helium concentration of 4.167 at.% are highly localized. A reduction of nearly 8% in bulk modulus is captured in the UO_2_ calculation, leading to a nearly 8% reduction in the bulk modulus and amplifying the mechanical embrittlement effect of helium at high uranium contents.

### 3.5. Linear Thermal Expansion Coefficient

**Figure 5:**
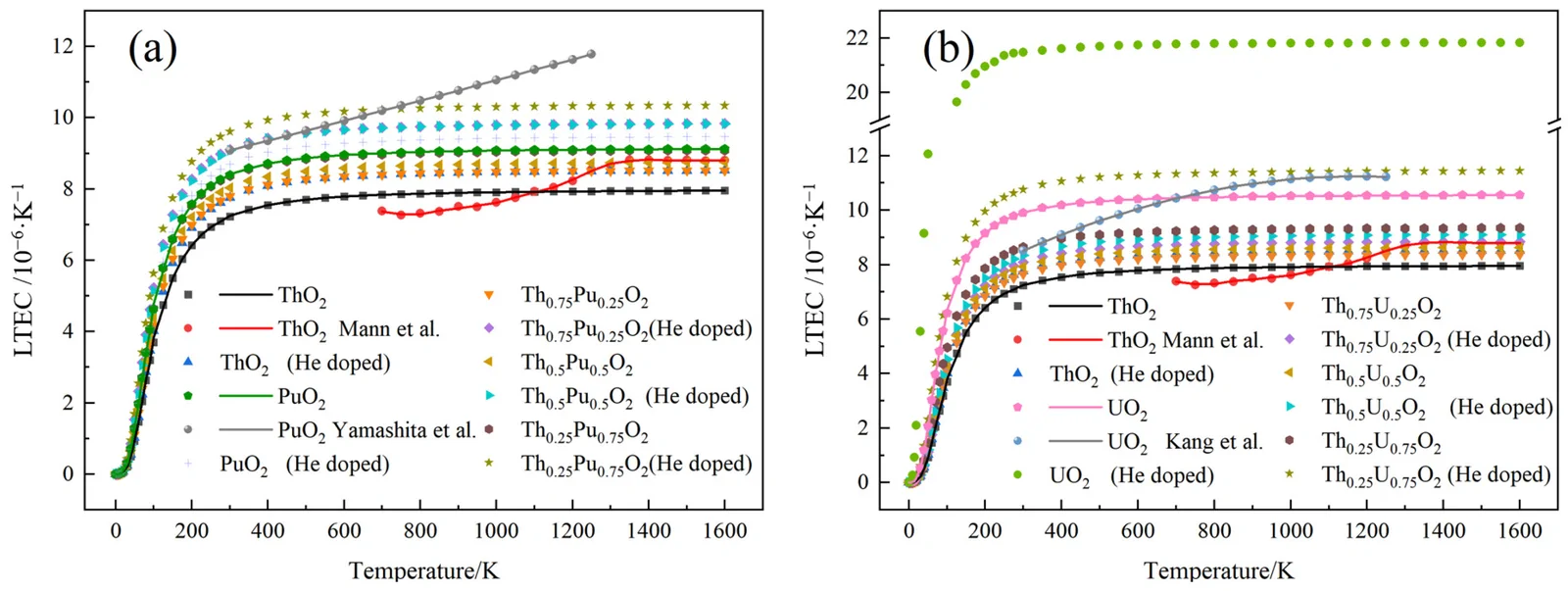
Phonon-QHA-predicted mean linear thermal expansion coefficients of the mixed oxides before and after He incorporation. (**a**) LTEC of (Th_1−x_Pu_x_)O_2_ before and after He doping [87,88]; (**b**) LTEC of (Th_1−x_U_x_)O_2_ before and after He doping [90].

The linear thermal expansion coefficient (LTEC, α_L_) reflects the relative rate of change of a material’s length under a unit temperature change. It is one of the important factors for evaluating the safety, thermal conductivity, and lifespan of a reactor. As shown in Figure 5, the linear thermal expansion coefficients of the two MOX fuels tend to saturate in the temperature range of 700 K to 1400 K, almost showing a linear behavior. The average linear expansion coefficient of ThO_2_ is 7.91 × 10^−6^ K^−1^ in the aforementioned temperature range, which is surprisingly in excellent agreement with the experimental value of 8.10 × 10^−6^ K^−1^ obtained by Mann et al. [87]. However, the LTEC of PuO_2_ deviates markedly from the experimental data measured via X-ray diffraction by Yamashita et al. [88]. Such a difference is attributable to the high-temperature properties predicted by the quasi-harmonic approximation (QHA), which are substantially lower than the measured values. Since the thermodynamic properties of actinide dioxides are highly analogous, a similarly large difference is observed in the LTEC of NpO_2_ calculated by Maldonado et al. [89]. For comparison, the reference LTEC data for UO_2_ reported by Kang et al. [90] are also included in Figure 5b.

To explore the effect of helium incorporation on the thermodynamic properties of the two MOX fuels, we specifically focus on the differences in the LTEC due to helium incorporation. ThO_2_ exhibits the most stable thermal expansion response. Similar to the trends observed in mechanical properties, the LTEC of both MOX fuels vary in an interpolative manner with the thorium content. Helium incorporation enhances the sensitivity of lattice volume to temperature, particularly in UO_2_, where α_l_ increases from 10.55 × 10^−6^ to 21.83 × 10^−6^ K^−1^. This nearly twofold increase indicates that He significantly contributes to the temperature dependence of the UO_2_ lattice parameters, demonstrating that helium exerts the strongest impact on the thermal expansion behavior of uranium-based nuclear fuels.

### 3.6. Migration Paths of Helium in Thorium Dioxide, Plutonium Dioxide, and Uranium Dioxide

**Figure 6:**
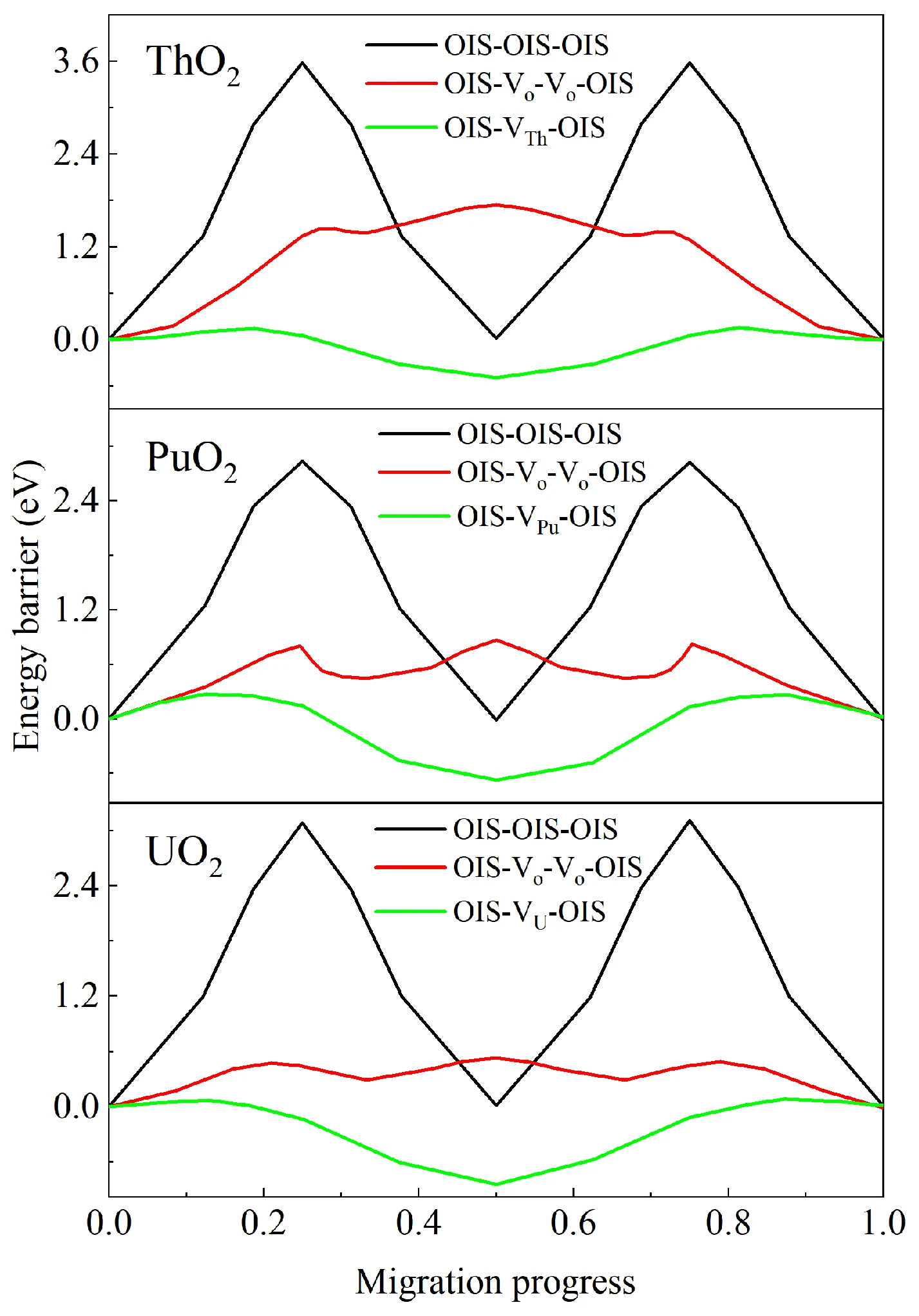
Transition state energy of a single He atom pass through 2 × 2 × 2 ThO_2_, PuO_2_ and UO_2_ supercells.

The effect of helium on the ThO_2_ structure depends not only on its incorporation energy but also on the minimum energy path for its diffusion [34]. Figure 6 shows the migration energy barrier curves of a single He atom at the OIS, metal and oxygen defect positions in ThO_2_, PuO_2_ and UO_2_. The migration trend of He in a given pathway is observed to be similar in all three nuclear fuels. However, the migration barrier of helium at the OIS of ThO_2_ is 3.58 eV, which is significantly higher than that of PuO_2_ and UO_2_, which are 2.82 and 3.08 eV, respectively. This is also indicative of the low diffusion rate of He in ThO_2_. In addition, the metal vacancies in the three oxides act as trap sites for He atoms. Among the migration pathways involving OISs, metal vacancies, and oxygen vacancies, helium migration between adjacent oxygen vacancies has the lowest energy barrier (0.42 eV). This implies that helium will migrate between adjacent oxygen vacancies. The helium atoms trapped in the O defect sites are not as tightly bound as those in the metal defects. In this case, the helium atoms may return to the crystal matrix, which is the re-solution phenomenon.

## 4. Conclusions

In this study, the effects of helium incorporation on the structural, electronic, mechanical, and thermodynamic properties of (Th_1−x_Pu_x_)O_2_ and (Th_1−x_U_x_)O_2_ MOX were systematically investigated using first-principles GGA + U calculations. The migration behavior of helium in ThO_2_, PuO_2_, and UO_2_ was further examined under different local defect environments. After structural relaxation, the fluorite framework was retained for all investigated compositions, although slight tetragonal or orthorhombic distortions were induced by magnetic ordering, cation substitution, and helium incorporation. The lattice parameters and average metal–oxygen bond lengths followed Vegard-type compositional trends before and after helium incorporation, indicating good structural compatibility in both Th–Pu and Th–U MOX systems. The volumetric expansion caused by helium remained limited, with the maximum expansion below approximately 0.6%.

The calculated helium incorporation energies were positive for all systems. Helium accommodation was energetically less favorable in UO_2_. DOS analysis showed that the band gaps of both mixed oxide systems are smaller than those of pure ThO_2_. UO_2_ lost its insulating character at a helium concentration of 4.167 at.%.

The elastic properties showed an approximately interpolative dependence on composition. After helium incorporation, the bulk modulus of (Th_0.25_U_0.75_)O_2_ and UO_2_ was reduced by approximately 7.18 and 15.54 GPa, respectively, showing that U-rich systems are more sensitive to He-induced mechanical softening. The calculated LTEC of ThO_2_ in the range 700–1400 K agreed well with the experimental value. For UO_2_, helium incorporation increased LTEC in the range of 10.55 × 10^−6^–21.83 × 10^−6^ K^−1^, indicating a pronounced reduction in thermal dimensional stability.

Helium migration was strongly dependent on the local defect environment. The interstitial migration barrier was calculated to be 3.58 eV in ThO_2_, 2.82 eV in PuO_2_, and 3.08 eV in UO_2_, indicating that He diffusion is most strongly suppressed in ThO_2_. Metal vacancies acted as deep trapping sites for He, whereas migration between adjacent oxygen vacancies required a maximum barrier of only 0.42 eV. Therefore, oxygen-vacancy networks may provide low-barrier diffusion paths, while metal vacancies may serve as stable He trapping centers. Overall, increasing the ThO_2_ fraction was shown to improve the structural, mechanical, and thermal resistance of MOX fuels to He-induced degradation.

## Figures and Tables

**Table 1:** The incorporation energy of a He atom into (Th_1−x_Pu_x_)O_2_ and (Th_1−x_U_x_)O_2_ mixed oxides.

Incorporation Energy/eV	x = 0	x = 0.25	x = 0.5	x = 0.75	x = 1
E[(Th_1−x_Pu_x_)O_2_]	0.84	0.87	0.92	0.98	1.02
E[(Th_1−x_U_x_)O_2_]		0.86	0.96	1.23	1.52

**Table 2:** Band gaps of (Th_1−x_Pu_x_)O_2_ and (Th_1−x_U_x_)O_2_ mixed oxides before and after He atom doped.

(Th_1−x_Pu_x_)O_2_/eV			**x = 0**	**x = 0.25**	**x = 0.5**	**x = 0.75**	**x = 1**
Band gap	Ideal	Present work	4.74	2.00	1.96	1.90	1.80
		Theoretical value	4.63–4.76 [45,48,65,66]	-	-	-	1.50–1.65 [18,67]
		Experimental value	5.9 [68,69]	-	-	-	1.80 [70]
	He doped	Present work	4.73	1.74	1.72	1.85	1.73
(Th_1−x_U_x_)O_2_							
Band gap	Ideal	Present work		2.89	2.71	2.37	2.06
		Theoretical value		-	-	-	1.87–2.06 [71,72,73]
		Experimental value		-	-	-	2.10 [74]
	He doped	Present work		2.84	2.51	1.72	-

**Table 3:** The elastic constants of (Th_1−x_Pu_x_)O_2_ and (Th_1−x_U_x_)O_2_ (x = 0, 0.25, 0.5, 0.75, 1) before and after He atom doped.

MOX	Elastic Constants/GPa					
	C_11_ (C_22_)	C_33_	C_12_ (C_13_)	C_23_	C_44_ (C_55_)	C_66_
**ThO_2_**						
GGA + U (present work)	344.33		106.12		80.27	
GGA + U [45]	344.3		106.0		73.2	
GGA [48]	349.5		111.4		70.6	
GGA [75]	352.2		107.8		74.6	
GGA [76]	346.11		117.64		88.47	
Experimental value [77]	367		106		79	
He-ThO_2_	342.25 (342.41)	342.36	105.68 (105.65)	105.65	79.75 (79.52)	79.51
**PuO_2_**						
GGA + U (present work)	382.47 (368.23)	361.51	114.06 (110.84)	110.61	71.38 (77.63)	69.75
GGA + U [45]	372.70 (372.90)	378.70	109.40 (109.80)	110.00	69.20 (69.00)	68.10
GGA + U [20]	369 (369)	378	109 (111)	112	71 (65)	67
GGA + U [78]	367.21		108.99		57.93	
He-PuO_2_	376.88 (363.44)	363.48	111.36 (111.36)	112.90	72.92 (77.22)	70.12
**(Th_1_** **_−_** **_x_** **Pu_x_)O_2_**						
(Th_0.75_Pu_0.25_)O_2_	344.98 (349.57)	349.25	105.06 (106.19)	106.71	75.65 (75.34)	76.18
He-(Th_0.75_Pu_0.25_)O_2_	299.80 (261.16)	347.53	119.61 (127.80)	138.72	72.16 (71.49)	70.48
(Th_0.5_Pu_0.5_)O_2_	360.83 (352.57)	357.59	110.95 (109.19)	105.66	73.83 (74.00)	72.13
He-(Th_0.5_Pu_0.5_)O_2_	356.61 (173.05)	341.91	98.35 (54.42)	173.05	69.23 (71.58)	60.72
(Th_0.25_Pu_0.75_)O_2_	362.73 (357.17)	362.35	110.90 (108.88)	108.91	67.91 (70.94)	73.14
He-(Th_0.25_Pu_0.75_)O_2_	268.69 (338.26)	339.63	166.85 (146.31)	69.31	70.86 (72.54)	71.70
**UO_2_**						
GGA + U (present work)	364.21 (374.31)	374.77	110.19 (110.59)	102.50	63.68 (64.26)	64.08
GGA + U [45]	361.8	368.8	109.4	107.7	63.0	63.3
GGA + U [79]	378.3		110.3		63.7	
GGA + U [80]	354.24		111.03		57.69	
Experimental value [81]	389		119		60	
He-UO_2_	362.82 (370.53)	368.65	89.44 (88.25)	81.30	48.27 (53.74)	56.21
**(** **Th** **_1−x_** **U_x_)O_2_**						
(Th_0.75_U_0.25_)O_2_	349.90 (348.40)	348.41	106.94 (106.94)	107.04	72.92 (78.78)	78.78
He-(Th_0.75_U_0.25_)O_2_	347.91 (345.99)	346.03	106.57 (106.57)	106.60	73.53 (73.86)	73.87
(Th_0.5_U_0.5_)O_2_	355.96 (352.65)	353.04	107.54 (107.58)	109.05	65.84 (73.51)	73.48
He-(Th_0.5_U_0.5_)O_2_	353.95 (350.51)	349.62	107.69 (108.10)	109.79	64.19 (73.08)	72.46
(Th_0.25_U_0.75_)O_2_	356.89 (355.30)	335.84	104.38 (113.48)	119.78	56.39 (61.89)	69.91
He-(Th_0.25_U_0.75_)O_2_	325.89 (322.26)	330.41	106.05 (131.94)	110.04	57.06 (60.23)	46.19

## Data Availability

The original contributions presented in this study are included in the article/Supplementary Materials. Further inquiries can be directed to the corresponding authors.

## Supplementary Materials

The following supporting information can be downloaded at: https://www.mdpi.com/article/10.3390/ma19183828/s1, File S1: All raw data supporting the figures and tables presented in this study, together with density-of-states (DOS) plots of (Th_1−x_Pu_x_)O_2_ and (Th_1−x_U_x_)O_2_ before and after He incorporation.

## Abbreviations

The following abbreviations are used in this manuscript:

DFT	density functional theory
MOX	mixed oxide
PWRs	pressurized water reactors
AFM	antiferromagnetic
OIS	octahedral interstitial site
FPs	fission products
PAW	projector-augmented wave
V_m_	metal vacancies
V_o_	oxygen vacancies
VASP	Vienna Ab initio Simulation Package
GGA	generalized gradient approximation
PBE	Perdew–Burke–Ernzerhof
SOC	spin–orbit coupling
QHA	quasi-harmonic approximation
CI-NEB	climbing nudged elastic band
TDOS	total density of states
PDOS	partial density of states
B	bulk modulus
LTEC	linear thermal expansion coefficient

## Appendix A

The convergence of the total energy of the ThO_2_ system was examined with respect to two key computational parameters—the plane-wave cutoff energy and the k-point mesh—using the VASP package. For the 2 × 1 × 1 supercell, total energy was first calculated at cutoff energy values ranging from 300 to 800 eV in increments of 50 eV, while a fixed 6 × 12 × 12 *k*-point mesh was employed. Subsequently, the *k*-point convergence was tested at cutoff energy = 550 eV using 1 × 2 × 2, 2 × 4 × 4, 3 × 6 × 6, 4 × 8 × 8, 5 × 10 × 10, and 6 × 12 × 12 meshes. The results are presented in Figure A1. Although total energy exhibits a non-monotonic dependence on cutoff energy at relatively low cutoff energies, its variation becomes substantially smaller above 550 eV. From 550 to 800 eV, the total energy variation is approximately 0.005 eV per supercell, corresponding to about 0.2 meV per atom for the 24-atom supercell. Therefore, cutoff energy = 550 eV was selected as a practical compromise between computational accuracy and cost. At this cutoff energy, total energy decreases markedly when the *k*-point mesh is increased from 1 × 2 × 2 to 2 × 4 × 4, whereas further increasing the mesh to 6 × 12 × 12 changes total energy by less than 0.001 eV. Consequently, a 4 × 8 × 8 k-point mesh was adopted for the subsequent calculations of the 2 × 1 × 1 ThO_2_ supercell.

## References

[1] International Atomic Energy Agency (2025). Nuclear Power Reactors in the World, 2025 Edition.

[2] Abbott D. (2011). Is nuclear power globally scalable?. Proc. IEEE.

[3] Herring J.S., MacDonald P.E. Characteristics of Mixed Thorium-Uranium Dioxide High Burnup Fuel. Proceedings of the ANS 1999 Annual Meeting.

[4] Herring J.S., MacDonald P.E., Weaver K.D., Kullberg C. (2001). Low cost, proliferation resistant, uranium-thorium dioxide fuels for light water reactors. Nucl. Eng. Des..

[5] Zhao X., Driscoll M.J., Kazimi M.S. (2001). Micro-Heterogeneous Thorium Based Fuel Concepts for Pressurized Water Reactors.

[6] International Atomic Energy Agency (2009). Status of Minor Actinide Fuel Development.

[7] Ronchi C., Hiernaut J.P. (1996). Experimental measurement of pre-melting and melting of thorium dioxide. J. Alloys Compd..

[8] Fink J.K. (2000). Thermophysical properties of uranium dioxide. J. Nucl. Mater..

[9] International Atomic Energy Agency (2005). Thorium Fuel Cycle—Potential Benefits and Challenges.

[10] Hubert S., Purans J., Heisbourg G., Moisy P., Dacheux N. (2006). Local structure of actinide dioxide solid solutions Th_1−x_U_x_O_2_ and Th_1−x_Pu_x_O_2_. Inorg. Chem..

[11] International Atomic Energy Agency (2012). Role of Thorium to Supplement Fuel Cycles of Future Nuclear Energy Systems.

[12] Roudil D., Bonhoure J., Pik R., Cuney M., Jégou C., Gauthier-Lafaye F. (2008). Diffusion of radiogenic helium in natural uranium oxides. J. Nucl. Mater..

[13] Liu X.-Y., Andersson D.A. (2018). Revisiting the diffusion mechanism of helium in UO_2_: A DFT+U study. J. Nucl. Mater..

[14] Ge X., Jiang Y., Yu X., Zhang G., Shi Y., Cai B., Peng Q., Huang H. (2025). Preparation and Characterization of Graphene-Nanosheet-Reinforced Ni-17Mo Alloy Composites for Advanced Nuclear Reactor Applications. Materials.

[15] Yang F., Boulet P., Record M.-C. (2025). DFT Investigation of a Direct Z-Scheme Photocatalyst for Overall Water Splitting: Janus Ga_2_SSe/Bi_2_O_3_ Van Der Waals Heterojunction. Materials.

[16] Szałowski K. (2024). Janus Monolayer of 1T-TaSSe: A Computational Study. Materials.

[17] Dorado B., Garcia P. (2013). First-principles DFT+U modeling of actinide-based alloys: Application to paramagnetic phases of UO_2_ and (U,Pu) mixed oxides. Phys. Rev. B.

[18] Zhang P., Wang B.-T., Zhao X.-G. (2010). Ground-state properties and high-pressure behavior of plutonium dioxide: Density functional theory calculations. Phys. Rev. B.

[19] Yang Y., Wang B., Zhang P. (2013). Electronic and mechanical properties of ordered (Pu,U)O_2_ compounds: A density functional theory+U study. J. Nucl. Mater..

[20] Ghosh P.S., Arya A. (2019). First-principles study of phase stability, electronic and mechanical properties of plutonium sub-oxides. Phys. Chem. Chem. Phys..

[21] Kuganathan N., Ghosh P.S., Arya A., Grimes R.W. (2018). Helium trapping and clustering in ThO_2_. J. Nucl. Mater..

[22] Jegadeesan P., Amirthapandian S., Panigrahi B.K., Kaur G., Sanjay Kumar D., Magudapathy P., Ananthasivan K. (2018). Aggregation and ordering of helium in thoria. J. Alloys Compd..

[23] Shao K., Han H., Zhang W., Wang H., Wang C.-Y., Guo Y.-L., Ren C.-L., Huai P. (2017). First-principles study of noble gas stability in ThO_2_. J. Nucl. Mater..

[24] Shao K., Han H., Zhang W., Wang C.-Y., Guo Y.-L., Ren C.-L., Huai P. (2017). First-principles study of helium clustering at initial stage in ThO_2_. Chin. Phys. B.

[25] Rahman M.J., Szpunar B., Szpunar J.A. (2019). Comparison of thermomechanical properties of (U_x_,Th_1−x_)O_2_, (U_x_,Pu_1−x_)O_2_ and (Pu_x_,Th_1−x_)O_2_ systems. J. Nucl. Mater..

[26] Rahman M.J., Szpunar B., Szpunar J.A. (2020). Dependence of thermal conductivity on fission-product defects and vacancy concentration in thorium dioxide. J. Nucl. Mater..

[27] Blöchl P.E. (1994). Projector augmented-wave method. Phys. Rev. B.

[28] Chen J.-L., Kaltsoyannis N. (2022). DFT + *U* Study of Uranium Dioxide and Plutonium Dioxide with Occupation Matrix Control. J. Phys. Chem. C.

[29] Faber J., Lander G.H. (1976). Neutron diffraction study of UO_2_: Antiferromagnetic state. Phys. Rev. B.

[30] Kresse G., Furthmüller J. (1996). Efficiency of ab-initio total energy calculations for metals and semiconductors using a plane-wave basis set. Comput. Mater. Sci..

[31] Kresse G., Furthmüller J. (1996). Efficient iterative schemes for ab initio total-energy calculations using a plane-wave basis set. Phys. Rev. B.

[32] Perdew J.P., Burke K., Ernzerhof M. (1996). Generalized Gradient Approximation Made Simple. Phys. Rev. Lett..

[33] Kresse G., Joubert D. (1999). From ultrasoft pseudopotentials to the projector augmented-wave method. Phys. Rev. B.

[34] von Barth U., Hedin L. (1972). A local exchange-correlation potential for the spin polarized case. I. J. Phys. C Solid State Phys..

[35] Wen X.-D., Martin R.L., Roy L.E., Scuseria G.E., Rudin S.P., Batista E.R., McCleskey T.M., Scott B.L., Bauer E., Joyce J.J. (2012). Effect of spin-orbit coupling on the actinide dioxides AnO_2_ (An = Th, Pa, U, Np, Pu, and Am): A screened hybrid density functional study. J. Chem. Phys..

[36] Boettger J.C., Ray A.K. (2000). All-electron LCGTO calculations for uranium dioxide. Int. J. Quantum Chem..

[37] Boettger J.C., Ray A.K. (2002). Fully relativistic density functional calculations on hydroxylated actinide oxide surfaces. Int. J. Quantum Chem..

[38] Prodan I.D., Scuseria G.E., Martin R.L. (2006). Assessment of metageneralized gradient approximation and screened Coulomb hybrid density functionals on bulk actinide oxides. Phys. Rev. B.

[39] Prodan I.D., Scuseria G.E., Sordo J.A., Kudin K.N., Martin R.L. (2005). Lattice defects and magnetic ordering in plutonium oxides: A hybrid density-functional-theory study of strongly correlated materials. J. Chem. Phys..

[40] Liechtenstein A.I., Anisimov V.I., Zaanen J. (1995). Density-functional theory and strong interactions: Orbital ordering in Mott-Hubbard insulators. Phys. Rev. B.

[41] Ghosh P.S., Kuganathan N., Arya A., Grimes R.W. (2018). Phase stability, electronic structures and elastic properties of (U,Np)O_2_ and (Th,Np)O_2_ mixed oxides. Phys. Chem. Chem. Phys..

[42] Kuganathan N., Ghosh P.S., Galvin C.O.T., Arya A.K., Dutta B.K., Dey G.K., Grimes R.W. (2017). Fission gas in thoria. J. Nucl. Mater..

[43] Kuganathan N., Ghosh P.S., Arya A.K., Dey G.K., Grimes R.W. (2017). Energetics of halogen impurities in thorium dioxide. J. Nucl. Mater..

[44] Kotani A., Yamazaki T. (1992). Systematic analysis of core photoemission spectra for actinide di-oxides and rare-earth sesqui-oxides. Prog. Theor. Phys. Suppl..

[45] Ghosh P.S., Arya A. (2020). Structural, thermodynamic, electronic and elastic properties of Th_1−x_U_x_O_2_ and Th_1−x_Pu_x_O_2_ mixed oxides. Phys. Chem. Chem. Phys..

[46] Murray E., Zhou Y., Slater P., Smith R., Goddard P., Steele H. (2022). Atomistic simulation of helium diffusion and clustering in plutonium dioxide. Phys. Chem. Chem. Phys..

[47] Garrido F., Nowicki L., Sattonnay G., Sauvage T., Thomé L. (2004). Lattice location of helium in uranium dioxide single crystals. Nucl. Instrum. Methods Phys. Res. B.

[48] Wang B.-T., Shi H., Li W.-D., Zhang P. (2010). First-principles study of ground-state properties and high pressure behavior of ThO_2_. J. Nucl. Mater..

[49] Kanchana V., Vaitheeswaran G., Svane A., Delin A. (2006). First-principles study of elastic properties of CeO_2_, ThO_2_ and PoO_2_. J. Phys. Condens. Matter.

[50] Xiao H.Y., Zhang Y., Weber W.J. (2013). Thermodynamic properties of Ce_x_Th_1−x_ O_2_ solid solution from first-principles calculations. Acta Mater..

[51] Lu Y., Yang Y., Zhang P. (2012). Thermodynamic properties and structural stability of thorium dioxide. J. Phys. Condens. Matter.

[52] Sevik C., Çağın T. (2009). Mechanical and electronic properties of CeO_2_, ThO_2_, and (Ce,Th)O_2_ alloys. Phys. Rev. B.

[53] Idiri M., Le Bihan T., Heathman S., Rebizant J. (2004). Behavior of actinide dioxides under pressure: UO_2_ and ThO_2_. Phys. Rev. B.

[54] Olsen J.S., Gerward L., Kanchana V., Vaitheeswaran G. (2004). The bulk modulus of ThO_2_: An experimental and theoretical study. J. Alloys Compd..

[55] Colbourn E.A., Mackrodt W.C. (1983). The calculated defect structure of thoria. J. Nucl. Mater..

[56] Smith M.P., Davey T., Rushton M.J.D., Gilbert M.R., Middleburgh S.C. (2026). Discovery of magnetic superstructures and magnetic transition temperatures in UO_2_ and PuO_2_. J. Phys. Condens. Matter.

[57] Frazer B.C., Shirane G., Cox D.E., Olsen C.E. (1965). Neutron-diffraction study of antiferromagnetism in UO_2_. Phys. Rev..

[58] Pegg J.T., Shields A.E., Storr M.T., Wills A.S., Scanlon D.O., de Leeuw N.H. (2018). Hidden magnetic order in plutonium dioxide nuclear fuel. Phys. Chem. Chem. Phys..

[59] Pegg J.T., Shields A.E., Storr M.T., Wills A.S., Scanlon D.O., de Leeuw N.H. (2019). Magnetic structure of UO_2_ and NpO_2_ by first-principle methods. Phys. Chem. Chem. Phys..

[60] Gryaznov D., Heifets E., Sedmidubsky D. (2010). Density functional theory calculations on magnetic properties of actinide compounds. Phys. Chem. Chem. Phys..

[61] Vegard L. (1921). Die Konstitution der Mischkristalle und die Raumfüllung der Atome. Z. Phys..

[62] Gryaznov D., Rashkeev S., Kotomin E.A., Heifets E., Zhukovskii Y. (2010). Helium behavior in oxide nuclear fuels: First principles modeling. Nucl. Instrum. Methods Phys. Res. B.

[63] Crocombette J.-P. (2002). Ab initio energetics of some fission products (Kr, I, Cs, Sr and He) in uranium dioxide. J. Nucl. Mater..

[64] Gryaznov D., Heifets E., Kotomin E. (2009). Ab initio DFT+U study of He atom incorporation into UO_2_ crystals. Phys. Chem. Chem. Phys..

[65] Pegg J.T., Aparicio-Angles X., Storr M., de Leeuw N.H. (2017). DFT+U study of the structures and properties of the actinide dioxides. J. Nucl. Mater..

[66] Murphy S.T., Cooper M.W.D., Grimes R.W. (2014). Point defects and non-stoichiometry in thoria. Solid State Ion..

[67] Hernandez S.C., Holby E.F. (2016). DFT+U study of chemical impurities in PuO_2_. J. Phys. Chem. C.

[68] Griffiths T.R., Dixon J. (2000). Electron irradiation of single crystal thorium dioxide and electron transfer reactions. Inorg. Chim. Acta.

[69] Evans W.R., Barton S.C., Clemens M., Allred D.D. Understanding DC-bias sputtered thorium oxide thin films useful in EUV optics. Proceedings of the SPIE 6317, Advances in X-Ray/EUV Optics, Components, and Applications.

[70] McNeilly C.E. (1964). The electrical properties of plutonium oxides. J. Nucl. Mater..

[71] Chen Q., Lai X., Tang T., Bai B., Chu M., Zhang Y., Tan S. (2010). First-principles study of the electronic structure and optical properties of UO_2_. J. Nucl. Mater..

[72] Nerikar P., Watanabe T., Tulenko J.S., Phillpot S.R., Sinnott S.B. (2009). Energetics of intrinsic point defects in uranium dioxide from electronic-structure calculations. J. Nucl. Mater..

[73] Iwasawa M., Chen Y., Kaneta Y., Ohnuma T., Geng H.-Y., Kinoshita M. (2006). First-principles calculation of point defects in uranium dioxide. Mater. Trans..

[74] Schoenes J. (1978). Optical properties and electronic structure of UO_2_. J. Appl. Phys..

[75] Ghosh P.S., Arya A., Dey G.K., Kuganathan N., Grimes R.W. (2016). A computational study on the superionic behaviour of ThO_2_. Phys. Chem. Chem. Phys..

[76] Boudjemline A., Louail L., Islam M.M., Diawara B. (2011). Dependence of pressure on elastic, electronic and optical properties of CeO_2_ and ThO_2_: A first principles study. Comput. Mater. Sci..

[77] Macedo P.M., Capps W., Wachtman J.O. (1964). Elastic constants of single crystal ThO_2_ at 25 °C. J. Am. Ceram. Soc..

[78] Singh S., Sonvane Y., Nekrasov K.A., Kupryazhkin A.Y., Gajjar P.N., Gupta S.K. (2023). Effect of pressure on mechanical and optical properties of ThO_2_ and PuO_2_. J. Am. Ceram. Soc..

[79] Vazhappilly T., Pathak A.K. (2020). A first principle based study on the mechanical and thermal properties of UO_2_: Effect of La and Dy fission product concentrations. Comput. Mater. Sci..

[80] Singh S., Sonvane Y., Nekrasov K.A., Boyarchenkov A.S., Kupryazhkin A.Y., Gajjar P.N., Gupta S.K. (2022). Systematic investigation of electronic, mechanical and optical properties of UO_2_ at higher pressure: A DFT+U+SOC study. Solid State Sci..

[81] Fritz I.J. (1976). Elastic properties of UO_2_ at high pressure. J. Appl. Phys..

[82] Petit T., Freyss M., Garcia P., Martin P., Ripert M., Crocombette J.-P., Jollet F. (2003). Molecular modelling of transmutation fuels and targets. J. Nucl. Mater..

[83] Debelle A., Boulle A., Garrido F., Thomé L. (2011). Strain and stress build-up in He-implanted UO_2_ single crystals: An X-ray diffraction study. J. Mater. Sci..

[84] Guilbert S., Sauvage T., Erramli H., Barthe M.-F., Desgardin P., Blondiaux G., Corbel C., Piron J.P. (2003). Helium behavior in UO_2_ polycrystalline disks. J. Nucl. Mater..

[85] Guilbert S., Sauvage T., Garcia P., Carlot G., Barthe M.-F., Desgardin P., Blondiaux G., Corbel C., Piron J.P., Gras J.-M. (2004). He migration in implanted UO_2_ sintered disks. J. Nucl. Mater..

[86] Sattonnay G., Vincent L., Garrido F., Thomé L. (2006). Xenon versus helium behavior in UO_2_ single crystals: A TEM investigation. J. Nucl. Mater..

[87] Mann M., Thompson D., Serivalsatit K., Tritt T.M., Ballato J., Kolis J. (2010). Hydrothermal growth and thermal property characterization of ThO_2_ single crystals. Cryst. Growth Des..

[88] Yamashita T., Nitani N., Tsuji T., Inagaki H. (1997). Thermal expansions of NpO_2_ and some other actinide dioxides. J. Nucl. Mater..

[89] Maldonado P., Paolasini L., Oppeneer P.M., Forrest T.R., Prodi A., Magnani N., Bosak A., Lander G.H., Caciuffo R. (2016). Crystal dynamics and thermal properties of neptunium dioxide. Phys. Rev. B.

[90] Kang K.H., Ryu H.J., Song K.C., Yang M.S. (2002). Thermal expansion of UO_2_ and simulated DUPIC fuel. J. Nucl. Mater..

